# Re-Evaluating Stroke Systems of Care: Association of Transfer Status With Thrombectomy Outcomes at an Urban Comprehensive Stroke Center

**DOI:** 10.7759/cureus.16732

**Published:** 2021-07-29

**Authors:** Kainaat Javed, Andre Boyke, Ishan Naidu, Jessica Ryvlin, Joseph Dardick, Devikarani Kadaba, David J Altschul, Neil Haranhalli

**Affiliations:** 1 Neurological Surgery, Montefiore Medical Center Moses Campus, New York, USA; 2 Neurosurgery, Johns Hopkins Medical Institute, Baltimore, USA

**Keywords:** acute ischemic stroke, mechanical thrombectomy, systems of care, hub and spoke, transfer status, urban setting

## Abstract

Introduction

Given the efficacy of mechanical thrombectomies (MT) for large vessel occlusions (LVO), there is concern that the Hub and Spoke model of stroke care, which prioritizes initial assessment of the acute ischemic stroke (AIS) patient at a primary stroke center, would cause a delay in arterial reperfusion, thus leading to worse outcomes. In this study that occurred at our comprehensive stroke center in New York, we compared the clinical outcomes of patients that were either directly admitted for thrombectomy versus those who were transferred from another institution.

Methods

Retrospective review of the electronic medical record (EMR) was performed on all adult patients treated with endovascular therapy for ischemic stroke between January 2016 and February 2020. A bivariate analysis was performed to compare patients in the direct admit versus transfer group. A multivariable logistic regression model was developed to determine which factors affect 90-day modified Rankin score (mRS) and to evaluate if transfer status was an independent predictor in this model.

Results

Three hundred and twenty-five patients were included in this study; 127 patients belonged to the direct admit group while 198 were in the transfer group. Thirteen patients (20%) in the direct admit group had a 90-day mRS score of 0-2 and so did 29 patients (25.2%) in the transfer group; thus, no statistically significant difference found in clinical outcomes between both groups (p-value = 0.427). In a multivariable logistic regression model that accounts for age, gender, smoking status, baseline mRS, presenting National Institute of Health Stroke Scale (NIHSS), procedure duration, thrombolysis in cerebral infarction (TICI) score, post-NIHSS and decompressive hemicraniectomy, transfer status was not found to be predictive of clinical outcomes (OR 0.727 95% CI 0.349-1.516; p-value = 0.396).

Conclusion

Transfer status is not significantly associated with 90-day outcome. Since Hub and Spoke is not associated with worse outcomes compared to direct admit, it remains a viable model for providing effective care to stroke patients in an urban setting.

## Introduction

Stroke is the fifth leading cause of death in the United States and costs the healthcare system more than $46 billion every year [1]. 87% of all strokes are characterized as ischemic, in which an intracranial artery is occluded. Symptoms of ischemic stroke vary depending on the affected vasculature - notable symptoms may include hemiparesis, neglect, aphasia, and sensory deficits [2]. Because of the symptom severity and long-term neurological damage, time to treatment is imperative in acute ischemic stroke (AIS). Patients that are seen within three hours of symptom onset have less disability than those patients receiving delayed care greater than three hours after onset [3].

The two mainstays of stroke treatment are intravenous recombinant tissue plasminogen activator (tPA) and mechanical thrombectomy (MT) with a stent retriever. Both treatments have time constraints, as IV tPA is contraindicated if time from last known normal is greater than 4.5 hours, while MT is most supported if time from last known normal is greater than six hours [4]. One study showed that a time from onset to tissue-type plasminogen activator treatment (OTT) reduction of 60 minutes increased the probability of favorable outcome when using tPA by 94% [5]. Despite this favorable outcome, because of the time constraint and also the association with increased risk of intracranial hemorrhage, rigid criteria have been developed for the use of tPA in AIS that often preclude the use of this treatment. In 2015, trials such as MR CLEAN, EXTEND-IA, REVASCAT, SWIFTPRIME, ESCAPE, THRACE, and THERAPY demonstrated that for patients with large vessel occlusions (LVO), MT in addition to standard medical management resulted in improved patient outcomes, establishing MT as a new standard of care for treatment of AIS from LVO. The DAWN trial in 2018 even showed that for patients with last known well from six to 24 hours earlier, MT plus standard medical care had superior disability and functional outcomes at 90 days compared to standard medical care alone [6].

The emergence of the two main forms of treatment for AIS has led to the establishment of a tiered system for patient triage and treatment with the certification of primary stroke centers (PSC) and comprehensive stroke centers (CSC) based on the availability of medical services for the treatment of AIS. The first tier is the acute stroke-ready hospital (ASRH), characterized by rapid stroke assessment, stabilization, and administration of tPA, but without ability of prolonged stroke care and/or rehabilitation. After tPA treatment and stabilization at these centers, AIS patients may be transferred to a higher-level stroke center for more workup and management. The second tier is the PSC, which consists of an interdisciplinary stroke team, advanced imaging, and an inpatient stroke unit. The third level, thrombectomy capable stroke centers (TSC), have a neurointerventional team that can perform MT procedures and monitor patients in the neurological ICU. The highest tier is the CSC which is capable of delivering comprehensive diagnostic and treatment services to AIS patients. These services include a multidisciplinary team of providers capable of not only administering tPA and MT, but also managing complex cases and post-treatment complications including neurosurgical services. The cross-disciplinary collaboration of the CSC is what differentiates this level from TSC [7]. While this tiered system may be important for effective triage and resource allocation, there is some evidence supporting that CSCs are better suited for more rapid acute reperfusion than PSCs. These data highlight the question of how best to utilize these varied and tiered centers to most effectively treat patients with AIS and optimize outcomes. 

Along these lines, various strategies for the coordination of patient transport and care within the constraints of this tiered system have been proposed [8]. One proposed strategy, “Drip and Ship” or “Hub and Spoke”, recommends transporting patients to the closest stroke center for tPA, followed by a transfer to a higher tier stroke center, TSC or CSC, that can perform an MT if indicated. This strategy prioritizes patients receiving more rapid initial evaluation and medical management, possibly with IV tPA, at the expense of delaying time to MT, making use of the fact that there are often far more PSCs available compared to CSCs. Another strategy, “Direct to Mothership”, recommends transporting patients directly to a center capable of providing MT. This strategy prioritizes reducing time to MT at the expense of time to tPA, recognizing MT is now well-established as the goal treatment when possible. This strategy depends on the ability of EMS to recognize large vessel occlusion (LVO) strokes, a primary criterion for MT [7], and misclassification may lead to adverse outcomes from delay in tPA.

Prior studies have explored the differences in the two strategies of stroke management: (1) Hub and Spoke and (2) Mothership. A meta-analysis of 18 studies including over 7,000 patients found that the mothership model was associated with a superior functional independence at 90 days compared to the Hub and Spoke model [9]. However, not all studies demonstrated a discrete difference in outcomes when comparing the two strategies. A cohort study of the New York Statewide Planning and Research Cooperative System (SPARCS) database from 2009 to 2015 found that patients transferred to a CSC (Hub and Spoke) did not have a higher fatality rate or length of stay compared to those patients presenting for initial treatment (mothership) at the hospital [8]. Two additional studies also observed no significant difference in 90-day functional difference between the two groups [10,11]. Thus, there remains uncertainty and a need for further research regarding the most beneficial patient coordination strategy to minimize adverse patient outcomes in AIS.

Given the efficacy of MT for large vessel occlusions (LVO), there is concern that the Hub and Spoke model of stroke care, which prioritizes initial assessment of the AIS patient at a primary stroke center, would cause a delay in arterial reperfusion, potentially leading to worse outcomes. In this study, conducted at our comprehensive stroke in an urban setting (Bronx, NY), we aimed to address these questions and compare the clinical outcomes of patients who underwent MT and were either directly admitted versus those who were transferred from another institution.

## Materials and methods

Data collection and management

In this study, we identified 325 patients treated with mechanical thrombectomy for acute ischemic stroke between January 2016 and February 2020 at our comprehensive stroke center in the Bronx, NY. We used electronic medical records (EMR) to identify and include patients aged 18 years or older and excluded patients if their diagnosis did not include acute ischemic stroke or if their age was 18 years or younger at initial presentation. The index date for data collection was set as the date of treatment with mechanical thrombectomy. This study was conducted as a retrospective review of mechanical thrombectomy procedures following AIS.

We collected patient demographics, which included age, race, sex, and ethnicity, as well as common comorbidities such as coronary artery disease, hypertension, diabetes mellitus, peripheral vascular disease, and smoking status. Smoking status was classified as either never smoker, current smoker, or former smoker. Additional history collected included prior stroke history and previous use of dual antiplatelet therapy (DAPT).

Presenting stroke severity for each patient prior to MT was included by collecting baseline modified Rankin scale (mRS), presenting NIH Stroke Scale (NIHSS), and Alberta Stroke Program Early CT Score (ASPECTS). Site of occlusion was identified as either middle cerebral artery (MCA), anterior cerebral artery (ACA), internal carotid artery (ICA), or posterior circulation. Additional presenting patient information acquired included hospital transfer status, time of symptom onset, time of presentation to the emergency department, and use of intravenous tissue plasminogen activator (tPA) prior to MT.

Intraoperative data included in this study consisted of procedure length, number of passes, and type of anesthesia used. Anesthesia was classified as either general anesthesia or monitored anesthesia care (MAC). Intervention success following MT was recorded using the thrombolysis in cerebral infarction scale (TICI). Finally, postoperative complications were assessed based on presence of hemorrhagic conversion or need for decompressive hemicraniectomy. Final outcomes were measured by length of hospital stay, disposition, 90-day mortality, and 90-day modified Rankin Scale (mRS) dichotomized to <2 and >2.

Statistical analysis

All statistical analyses were performed using STATA 16 statistical software. We used bivariate analysis to compare patients who were directly admitted versus patients who were transferred based on demographics, comorbidities, initial clinical presentation, intraoperative specifics, postoperative complications, and final outcomes. Chi-squared tests were employed in comparing categorical variables, while Student’s t-test was used to compare numerical variables with a normal distribution, and otherwise Mann-Whitney U test. We built an adjusted multiple logistic regression model to identify the effect of transfer status on good short-term outcomes based on a 90-day mRS of 0-2.

## Results

Out of the 325 patients treated with mechanical thrombectomy at our institution over a four-year period, 127 patients arrived directly to our comprehensive stroke center and 198 patients were first evaluated at a primary stroke center and eventually transferred to our comprehensive center once they were deemed eligible candidates for thrombectomy.

On bivariate analysis (Table 1), there were differences between the direct admit and transfer cohorts at baseline. For starters, there was a difference in the racial composition of both groups (p-value = 0.001). In the direct admit group, there were 35.4% of patients who identified as African-Americans and Hispanic. Meanwhile, in the transfer group, the percentage of Africans-Americans and Hispanics was 26.3% and 21.2% respectively. Furthermore, the groups differed in their baseline comorbidities. 79.5% of patients in the direct admit group had hypertension compared to 64.6% of patients in the transfer group (p-value = 0.004). Additionally, eight patients (6.3%) in the direct admit had peripheral artery disease (PAD) while no patients in the transfer group did (p-value = 0.001). Both groups also differed in their clinical presentation. 84.3% of patients in the direct admit cohort presented with an NIHSS score of greater than 10 while only 74.7% of patients in the transfer group presented with a high NIHSS score (p-value = 0.042). Moreover, 75.6% of patients in the direct admit cohort presented within six hours of stroke onset but only 64.7% of transfers presented to our emergency department within six hours of symptom onset (p-value = 0.038).

**Table 1:** Descriptive characteristics of the patient population based on admission type. CAD: coronary artery disease; HTN: hypertension, DM: diabetes mellitus, PAD: peripheral artery disease; DAPT: dual anti-platelet therapy; mRS: modified Rankin scale; NIHSS: National Institute of Health Stroke Scale; MCA: middle cerebral artery; ICA: internal carotid artery; ACA: anterior cerebral artery; ASPECTS: Alberta Stroke Programme Early Computed Tomography Score, IV: intravenous; tPA: tissue plasminogen activator, TICI: thrombolysis in cerebral infarction score; DHC: decompressive hemi-craniectomy; HC: hemorrhagic conversion; LOS: length of stay.

Characteristics	Direct admit (n = 127)	Transfer (n = 198)	p-value
Age (years)	70 (61-79)	68 (59-78)	0.469
Gender (male)	59 (46.5%)	99 (50%)	0.533
Race			0.001
Black	45 (35.4%)	52 (26.3%)	
White	16 (12.6%)	54 (27.3%)	
Hispanic	45 (35.4%)	42 (21.2%)	
Other	10 (7.9%)	19 (9.6%)	
Unknown	11 (8.7%)	31 (15.7%)	
Prior Stroke	27 (21.3%)	31 (15.7%)	0.198
CAD	22 (17.3%)	27 (13.6%)	0.365
HTN	101 (79.5%)	128 (64.6%)	0.004
DM	50 (39.4%)	60 (30.3%)	0.092
PAD	8 (6.3%)	0 (0%)	0.001
Smoker			0.668
Never	73 (57.4%)	92 (46.5%)	
Current	18 (14.2%)	27 (13.6%)	
Former	24 (18.9%)	39 (19.7%)	
Prior DAPT use	8 (6.3%)	10 (5.1%)	0.631
Baseline mRS (0-2)	95 (74.8%)	154 (77.8%)	0.536
Presenting NIHSS (>10)	107 (84.3%)	148 (74.7%)	0.042
Site of occlusion			0.636
MCA	102 (80.3%)	150 (75.8%)	
ICA	14 (11.0%)	22 (11.1%)	
ACA	0 (0%)	1 (0.5%)	
Posterior	11 (8.7%)	24 (12.1%)	
ASPECTS (>6)	119 (93.7%)	190 (96%)	0.358
Symptom onset (<6 hours)	96 (75.6%)	128 (64.7%)	0.038
IV tPA	59 (46.5%)	76 (38.4%)	0.150
Procedure length (min)	117 (97 - 147)	132 (97 - 158)	0.562
Anesthesia (general)	21 (16.5%)	28 (14.2%)	0.569
No. of Passes (1)	66 (56.9%)	95 (51.1%)	0.195
TICI (3-2b)	103 (81.1%)	160 (80.8%)	0.947
Post NIHSS (>10)	77 (60.6%)	121 (61.1%)	0.931
DHC	8 (6.3%)	11 (5.6%)	0.789
HC	31 (24.4%)	41 (20.8%)	0.447
ICU LOS (days)	1 (0-3)	1 (0-3)	0.329
Hospital LOS (days)	9 (5-17)	9 (6-15)	0.917
90-day mRS (0-2)	13 (20.0%)	29 (25.2%)	0.427
90-day mortality	27 (21.3%)	54 (27.3%)	0.221

While there were differences in baseline characteristics, there were no differences observed between outcomes. 81.1% of patients in the direct admit group and 80.8% of patients in the transfer group achieved near-complete reperfusion after successful thrombectomy as indicated by a TICI score of 2B-3 (p-value = 0.947). The complication rates were similar among both groups. 24.4% of patients in the direct admit group and 20.8% of patients in the transfer group had a hemorrhagic conversion of ischemic stroke (p-value = 0.447). Around 6% of patients in both groups needed a decompressive hemicraniectomy for malignant edema and impending herniation (p-value = 0.789). The median length of ICU stay was one day (p-value = 0.329) and median length of hospital stay was nine days (p-value = 0.917) for both groups. 20% of patients in the direct admit group and 25.2% of patients in the transfer group had a 90-day mRS score of 0 to 2 (p-value = 0.427). Lastly, the mortality rate for the direct admit and transfer groups was 21.3% and 27.3%, respectively (p-value = 0.221).

A multiple logistic regression model (Table 2) was constructed to identify independent predictors of favorable modified Rankin score of 0-2 at 90 days. The model was adjusted for age, gender, history of prior stroke, diabetes mellitus, smoking status, baseline mRS, presenting NIHSS score, TICI score, post-procedural NIHSS score, procedure length and need for decompressive hemicraniectomy. Once these variables were accounted for, the admit status of the patient was not found to be a statistically significant predictor of functional outcomes (OR 0.727 95% CI 0.349-1.516, p-value = 0.396). Our results show that a baseline mRS of 0-2 is an independent predictor of good functional outcome (OR 20.51 95% CI 4.145-101.5, p-value=0.001). However, the predictors of poor outcome include being a current smoker (OR 0.228 95% CI 0.072-0.720, p-value = 0.011), longer procedure length (OR 0.987 95% CI 0.978-0.997, p-value = 0.014), post NIHSS score > 10 (OR 0.146 95% CI 0.064-0.316, p-value = 0.001) and requiring a decompressive hemicraniectomy (OR 0.032 95% CI 0.003-0.341, p-value = 0.004).

**Table 2:** Multiple logistic regression model for modified Rankin scale 0-2. DM: diabetes mellitus; mRS: modified Rankin scale; NIHSS: National Institute of Health Stroke Scale; TICI: thrombolysis in cerebral infarction scale; DHC: decompressive hemi-craniectomy.

Independent predictor	Adjusted OR (95% CI)	p-value
Transfer status	0.727 (0.349-1.516)	0.396
Age	0.976 (0.953-1.000)	0.053
Gender (male)	1.761 (0.834-3.715)	0.137
Prior stroke	0.505 (0.160-1.586)	0.242
DM	0.609 (0.270-1.374)	0.233
Smoker (current)	0.228 (0.072-0.720)	0.011
Smoker (former)	1.128 (0.463-2.743)	0.791
Baseline mRS (0-2)	20.51 (4.145-101.5)	0.001
Presenting NIHSS (>10)	0.559 (0.222-1.403)	0.216
Procedure length (mins)	0.987 (0.978-0.997)	0.014
TICI (3-2b)	3.128 (0.797-12.28)	0.102
Post NIHSS (>10)	0.146 (0.064-0.316)	0.001
DHC	0.032 (0.003-0.341)	0.004

## Discussion

**Figure 1:**
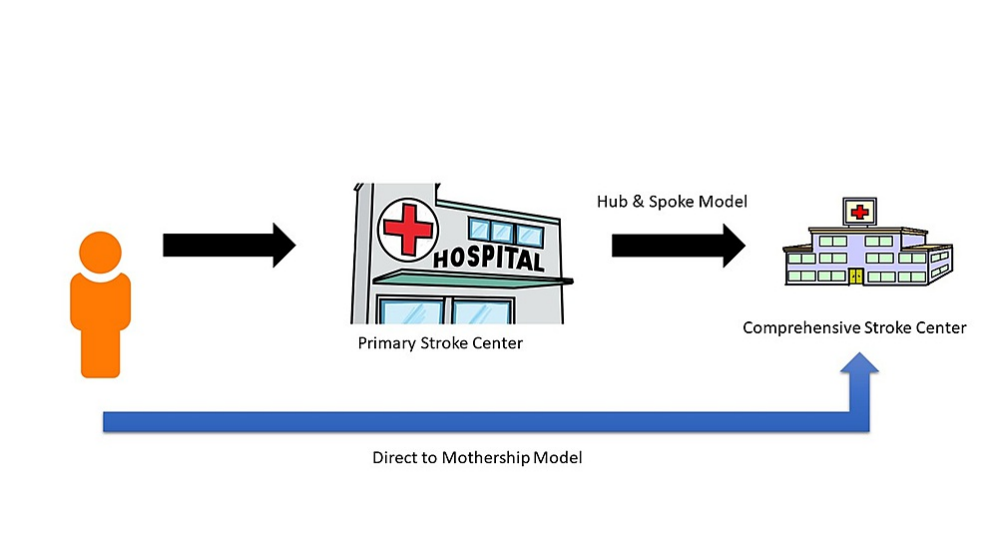
Models for triage of ischemic stroke patients. The two main models that we compared are the Hub & Spoke model and the Direct to Mothership model. In the Hub & Spoke model, patients are first taken to a primary stroke center while in the Direct to Mothership model, they are immediately taken to a comprehensive stroke center.

There are various models that are described in the literature for acute ischemic stroke triage for patients brought in by Emergency Medical Services (EMS). Two of these models include the “Hub and Spoke” model and the “Direct to Mothership” model as seen in Figure 1. These models differ primarily in regards to the type of stroke center patients should be transported to once a stroke call is activated to EMS. In the Hub and Spoke model, patients with AIS are transported to the closest center, whether it be a PSC or a CSC. In the Direct to Mothership model, AIS patients, especially those who are suspected of having an LVO, are preferentially transported to a CSC. In the literature, there is no clear consensus on which triage model is associated with the best functional outcome.

The stroke triage system that is used in New York City (NYC) is set forth by the NYC Regional Emergency Medical Advisory Committee (REMAC) [12]. In this model, when EMS is notified of a possible stroke call, they will use a clinical scale known as the Los Angeles Motor Scale (LAMS) to assess the patient. The LAMS scale is a 5-point scale. A score of 4 or 5 indicates that an LVO is likely and the patient is taken to a facility with endovascular capabilities unless the patient has an urgent need for medical attention. If the LAMS score is low (0-3), which is often the case, the patient is transported to the nearest center, which may be a PSC or CSC. If the patient’s last well-known time is greater than five hours or if the CSC is located more than 30 minutes away for a patient with a LAMS of 4-5, the patient is taken to a PSC. Thus, in essence, the stroke triage system that is currently set in place in NYC is consistent with the Hub & Spoke Model. 

Thus, the patients who made up our transfer group were patients with AIS due to LVOs who were initially taken to a PSC and were then transferred to our institution when their need for MT became apparent, as seen traditionally in the Hub and Spoke Model. The patients who made up the direct admit group were likely patients who brought by EMS to our CSC which fortunately happened to be the closest located hospital. Or if there were two hospitals that were located within close proximity to the patient’s location, one of them being a PSC and the other a CSC, and the patient was thought to have an LVO, transport to the CSC was preferred due to easy access to both thrombolytic therapy and thrombectomy. Thus, essentially, patients in our direct admit group were also triaged using the Hub and Spoke Model. However, a unique advantage of an urban setting such as NYC is that it is densely packed with multiple medical centers and when the Hub and Spoke model is implemented, sometimes a higher tiered facility such as a CSC becomes the primary institution that patients are evaluated at. 

There were differences in the baseline characteristics between both groups. Patients in the direct admit group were more likely to belong to a racial/ethnic minority, have comorbidities such as HTN & PAD and have a higher presenting NIHSS score. There are several possible explanations for these findings. For starters, if a patient has a higher score on a clinical scoring system such as LAMS, it raises suspicion for LVO and the patient is transported to a CSC. A higher score on LAMS score is associated with a higher NIHSS score usually [13]. Higher presenting NIHSS, which indicates a severe stroke, is seen in patients with baseline comorbidities [14]. Thus, it is possible that patients with baseline comorbidities and severe strokes, as measured by NIHSS, were preferentially transported to the CSC which is why they make up a greater percentage of the direct admit group. Another explanation for this finding is that it is reflective of the community that our CSC serves. In the neighborhood surrounding our hospital in the Bronx, there is a high percentage of patients who identify as racial minorities and have comorbidities at baseline [15,16]. Thus, it is possible that these patients either came to or were brought by EMS to our center since it was the closest hospital and they seek the majority of their care here.

There was also a difference in the timing from symptom onset till presentation to the CSC Emergency Room (ER). Patients in the transfer group were less likely to present within six hours than the direct admit group. This is likely because patients in the transfer group were, by definition, seen at another hospital first which delayed their presentation to our ER.

Regardless of how patients arrived at the CSC, the outcomes after thrombectomy between both groups were similar. There was no difference in clinical outcomes at 90-days and 90-day mortality rates. Furthermore, on logistic regression, being a transfer patient to CSC was not an independent predictor of 90-day outcomes. In the transfer group, patients were evaluated for early thrombolytic therapy at a PSC. While there was no difference in tPA administration between both groups, the patients in the transfer group likely received tPA earlier. Since the current American Heart Association (AHA) guidelines recommend bridging therapy [17], which includes the prompt delivery of IV thrombolytics for eligible AIS patients followed by endovascular thrombectomy, perhaps there is benefit to early administration of IV tPA that makes up for the delay in thrombectomy treatment. This theory is not directly supported by our data but is one possible explanation for comparable outcomes. More importantly, as demonstrated by the seminal DAWN and DEFUSE 3 trials [6,18], the initial window period for thrombectomy in AIS patients may be extended past the six-hour mark while still resulting in favorable outcomes. Our results suggest that in transfer patients who were initially evaluated at a PSC prior to undergoing thrombectomy, there was not a significant enough delay in EVT treatment to have an impact on functional outcomes.

Our findings suggest that in a densely packed urban setting, where fast access to PSCs and CSCs may be comparable and transport between the two is relatively short, the Hub and Spoke model may still be an effective system of stroke care. Patients in our study who were triaged using this model experienced the same outcomes as patients who were preferentially transported to a CSC or who happened to live close to a CSC. They did not experience a delay in arterial reperfusion and have worse outcomes which is the biggest criticism of the Hub and Spoke model. It is important to note that the literature is divided on this topic. Our results are consistent with other studies that reported no difference in outcomes between direct admit and transfer groups. [8] However, there are some studies that found that the direct to mothership model was associated with better outcomes. We believe that this discrepancy in the literature may be explained in part due to the setting in which the study is conducted. In a meta-analysis performed by Romoli et al, a few studies conducted at large tertiary centers located in urban areas with densely populated hospitals showed no difference in outcomes between both stroke systems of care models while a study conducted in rural Bavaria showed the direct admission to a CSC yielded better outcomes. [9] We realize that our findings are specific to our center located in New York City; at our location, either of the two stroke systems of care models may be implemented without leading to a change in outcomes for our patients. It is also interesting to note that in the literature, for studies that demonstrated that the direct admission to a CSC led to superior outcomes, there was a significant delay in the transfer times using the Hub and Spoke model. Probability modeling case studies have shown that if the transfer times are improved, that may lead to comparable outcomes among patients treated with both models of stroke care.

There are several limitations to our study. For patients who were not brought to our stroke center using EMS, a variety of factors may have influenced their decision to present to our institution including location of primary care provider, past experiences at a given center and desire for proximity to a certain location. In essence, these patients were not technically triaged using the Hub & Spoke model or another model of stroke care and yet were still included in the study. For patients who were brought by EMS, pre-hospital care data including the patient's site of origin, LAMS score in the field, and underlying reason for ultimate transfer to our facility was not evaluated in our analysis. This was due to a large number of missing data. When prehospital care data is not directly available, part of it may be extracted from triage nursing and ED provider notes. In our case, those notes were also not accessible, especially for a majority of the older cases. Active efforts are currently underway to recover that data and we hope to include it in our future projects. In theory, we could have performed an analysis using a more robust data set that included the mode of transport and the field assessment for each patient. However, the reason why we decided to proceed with this study despite the missing, aforementioned variables was due to the main purpose of our research. The purpose of this study was not to comment on the model of stroke triage that is in place in NYC or its long-term efficacy. The purpose of this study was to illustrate that when comparing two groups of AIS patients who, to a degree, may have been randomized by chance and mainly differ in regards to the tier of the stroke center that they were initially assessed at, but went on to receive the same life-saving treatment (i.e. thrombectomy), the 90-day functional outcomes are comparable. One population who we did not assess include patients who were transferred to our center and were no longer eligible for thrombectomy. They may be a possible source of significant morbidity and mortality and additional studies are needed to understand how the outcomes for this patient cohort influence developing models of stroke triage if at all. 

## Conclusions

In a single-center retrospective study that was conducted at our comprehensive stroke center in the Bronx, there were no differences in 90-day functional outcomes between patients who were directly admitted to our institution compared to patients who were transferred from a primary stroke center. Our findings suggest that in our city, the Hub & Spoke model may still be a viable model for stroke systems of care. While there are discrepancies among the studies in the literature on this topic, we believe that the difference in results is due to individual factors such as location and transport mechanisms that are set in place at each center which influence how efficient the implementation of the Hub & Spoke model is.
